# Developing the principles of chair based exercise for older people: a modified Delphi study

**DOI:** 10.1186/1471-2318-14-65

**Published:** 2014-05-19

**Authors:** Katie R Robinson, Paul Leighton, Philippa Logan, Adam L Gordon, Kevin Anthony, Rowan H Harwood, John RF Gladman, Tahir Masud

**Affiliations:** 1 Healthcare for Older People, Nottingham University Hospitals Trust, Queens Medical Centre, NG7 2UH Nottingham, UK; 2 Division of Rehabilitation and Ageing, Medical School, The University of Nottingham, Queens Medical Centre, NH7 2UH Nottingham, UK; 3 Division of Primary Care, Medical School, The University of Nottingham, NG7 2UH Nottingham, UK; 4 Falls and Bone Health Service, Nottingham CityCare Partnership, Newbrook House, 385 Alfreton Road, NG7 5LR Nottingham, UK

**Keywords:** Chair based exercise, Older people, Physical activity, Consensus development

## Abstract

**Background:**

Chair based exercise (CBE) is suggested to engage older people with compromised health and mobility in an accessible form of exercise. A systematic review looking at the benefits of CBE for older people identified a lack of clarity regarding a definition, delivery, purpose and benefits. This study aimed to utilise expert consensus to define CBE for older people and develop a core set of principles to guide practice and future research.

**Methods:**

The framework for consensus was constructed through a team workshop identifying 42 statements within 7 domains. A four round electronic Delphi study with multi-disciplinary health care experts was undertaken. Statements were rated using a 5 point Likert scale of agreement and free text responses. A threshold of 70% agreement was used to determine consensus. Free text responses were analysed thematically. Between rounds a number of strategies (e.g., amended wording of statements, generation and removal of statements) were used to move towards consensus.

**Results:**

16 experts agreed on 46 statements over four rounds of consultation (Round 1: 22 accepted, 3 removed, 5 new and 17 modified; Round 2: 16 accepted, 0 removed, 4 new and 6 modified; Round 3: 4 accepted, 2 removed, 0 new and 4 modified; Round 4: 4 accepted, 0 removed, 0 new, 0 modified).

Statements were accepted in all seven domains: the definition of CBE (5), intended users (3), potential benefits (8), structure (12), format (8), risk management (7) and evaluation (3).

The agreed definition of CBE had five components: 1. CBE is primarily a seated exercise programme; 2. The purpose of using a chair is to promote stability in both sitting and standing; 3. CBE should be considered as part of a continuum of exercise for frail older people where progression is encouraged; 4. CBE should be used flexibly to respond to the changing needs of frail older people; and 5. Where possible CBE should be used as a starting point to progress to standing programmes.

**Conclusions:**

Consensus has been reached on a definition and a set of principles governing CBE for older people; this provides clarity for implementation and future research about CBE.

## Background

For community dwelling populations there is clear evidence to support the hypothesis that exercise improves health and quality of life [1]. In older frail people, these programmes have a significant impact on reducing the risk and rate of falls [2] with an associated reduction in morbidity, mortality and costs to health and social care. Many exercise programmes are performed in standing and unaided [3]. Such programmes can be difficult or impossible for those who are immobile or very unstable and chair based exercise (CBE) may be offered in this context [4].

A recent systematic literature review considering CBE in older people [5] found many studies of poor methodological quality with small sample sizes. There was considerable variability in the outcome measures used, precluding pooled analysis. A key finding from this review was a lack of clarity over the definition of CBE, the purpose of CBE and the expected benefits of CBE for older people. There is therefore a lack of clarity regarding when practitioners should use CBE and what outcomes they should expect when they do so. This uncertainty also makes research to evaluate CBE difficult by virtue of a lack of central hypothesis about its role. Establishing a core set of principles which include a definition, purpose, delivery and expected outcomes will help to guide practitioners and further research.

Against this background, this study set out to establish expert consensus on what defining what CBE is, what the essential components of a CBE intervention should be and what benefits might be expected to result from a CBE programme.

## Methods

### Delphi process

In the absence of a strong evidence base and clear guidance for clinical practice a consensus development technique may provide a basis for decision making and further guidance [6]. The Delphi technique is a well-recognised consensus method used to determine the extent of agreement on an issue [7]. It is an iterative process which supports the development of expert consensus on a particular topic and is characterised by several aspects: anonymity, multiple iterations of a similar survey tool, feedback between rounds and statistical assessment of consensus scoring [6-9]. It involves a panel of experts undertaking a series of rounds to identify, clarify, refine and gain consensus [9].

### Framework for the Delphi process

It is acknowledged that the first round of a traditional Delphi process uses open questioning to identify the focus of the process [10]. Modifying the Delphi approach is however considered appropriate to ensure that that methodology is appropriate for the study aims rather than shaping the study aims to fit the methodology. In this study we used the following modified methods to develop the framework for the formal consensus process:

• Telephone consultations with stakeholders to explore key areas of chair based exercise

• Generation and discussion of structured statements at a team workshop of the study management group (all the co-authors)

Telephone interviews were used as an open data collection method which was then formalised in a team workshop. This approach was chosen to manage the breadth of open data anticipated in this consultation process and then allow formal consensus development in a manageable pragmatic way.

The focus and scope of the consensus process was determined through a review of current literature [5] and consultation with 11 stakeholders [2 academic geriatricians, 4 exercise physiologists/scientists, 2 physiotherapists representing AGILE, 2 exercise instructors, 1 physiotherapist delivering CBE]. Consultation comprised of telephone interviews with open questioning about key areas around chair based exercise. Seven key themes emerged from these consultations including defining CBE, potential benefits, intended users’ delivery and training. Thematic analysis was used to analyse the free text from the interviews.

Statements for round 1 of the Delphi process were generated by the study management group in a team workshop, which comprised 7 clinical researchers (2 physiotherapists, 1 occupational therapist and 4 geriatricians) with an interest in rehabilitation of frail older people. Statements were constructed within the 7 key themes based on current literature and the telephone consultation process. Each statement was discussed at length by the study management group, all of whom agreed on the final set of 42 statements.

### Participants and recruitment

To reflect the complexity and variety of expertise involved in the delivery of CBE to older people a purposive sampling strategy was developed to recruit Delphi experts involved in CBE from a variety of different clinical and professional settings. It acknowledged that defining and selecting experts in a Delphi study is a challenging process and may require a pragmatic approach that is appropriate to the study aims [11]. For this study an expert was defined as anybody with knowledge and experience of chair based exercise programmes in a frail elderly population. However this was not limited to a clinical setting and included experts with an academic background, exercise instructors and experts working in the voluntary and social care sectors. Experts were selected from specialist professional groups (e.g., Chartered Society of Physiotherapy Older People Network-AGILE, British Geriatrics Society, College of Occupational Therapists Older People Specialist Section), leading providers in CBE programmes (e.g., Later Life Training, Extend), charitable organisations involved in supporting older people (e.g., Age UK) and prominent clinicians and academics identified through a review of the literature.

In total 25 UK experts in the field of CBE for older people were invited to take part by e-mail and provided a Participant Information Sheet. Experts were asked to provide a summary of their experience and expertise in the field prior to being included in the study. All experts that were willing to take part returned a completed consent form prior to the consensus development process taking part.

### Procedure

Electronic communication methods (in this case e-mail and the SurveyMonkey web-service) were used to ensure anonymity of all participants. Anonymity supports the generation of responses which are free from the bias introduced by the issues of group dynamics influencing face-to-face discussions [8,12]. Participants were given one week to complete each round with a reminder e-mail sent at one week and two weeks after this deadline. If a participant had not completed the round at this point they were excluded from further rounds of the process.

To ensure strong retention of expert involvement, an upper limit of four rounds of investigation was set in this review [8]. It is also acknowledged that having a planned number of rounds is an indicator of good quality in designing a Delphi study [13]. The four rounds of investigation took a total of 5 months.

Each survey tool consisted of a series of structured statements constructed so that responders could rate their level of agreement using a five point Likert scale. Open response questions were also used to allow participants to comment freely on each statement.

A Delphi study is an iterative process that uses repeated communication to refine expert opinion on a specific topic and move towards an accepted level of consensus. It was therefore necessary to analyse and review the findings of each round and revise the survey instrument for the following round. Procedures used in this process were:

• A summary of the panel scoring was presented for each statement in the following round. This panel scoring included the level of agreement of each statement along with a text summary of the comments given in the previous round. This provided a context for participants to inform their rescoring to the revised survey instrument.

• Any statement which reached consensus (agreement or disagreement) was removed from rescoring in further rounds of the survey.

• Statements where consensus was reached were modified where the free text comments indicated this was appropriate. Free text comments were analysed thematically and any themes that were not consistent with the statement were used to revise the statement. This statement was then rescored in the following round and participants were given the opportunity to provide open text comments.

• The wording of statements was modified where appropriate to reflect any comments from participants. Free text comments were analysed thematically and were then used to modify the statement. This statement was then presented back to the panel in the following round for rescoring and comments.

• New statements were formulated based on comments and when further clarification was needed.

There is no universally accepted threshold for defining consensus as part of the Delphi process, with thresholds for consensus ranging from 55%-100% in the published literature [7]. A heterogeneous group of expert was considered appropriate to allow for different perspectives to be explored [10]. A predefined consensus level is an indicator good quality Delphi research [7] and the consensus level is influence by the study aims [12]. A 70% threshold was considered appropriate for this study and is consistent with other research using a Delphi technique [14,15]. Statements were considered to be “principles governing CBE” once the 70% threshold for consensus was reached.

## Results

### Response rate

25 experts were invited to take part and 17 (68%) agreed to do so. A 100% response rate (n = 17) was achieved in Round 1 and a 94% response rate (n = 16) for Rounds 2, 3 and 4. Reasons for not taking part or completing the process included time constraints and a perceived lack of expertise in the area.

### Participants

Of the 25 experts invited 17 participants took part in the modified Delphi process. They represented a range of health care professions including 6 physiotherapists, 2 occupational therapists, 2 CBE exercise leaders, 1 rehabilitation consultant, 1 clinical exercise specialist, 1 older people’s lead at the BHF National Centre for Physical Activity and Health, 3 academics in the field of exercise for older people (one from a sport science, one from a physiology department and one from a nursing department) and 1 older persons specialist nurse. The experts worked in a range of settings including NHS acute and community services, private exercise training providers, social care, academic institutions and The Care Inspectorate, Scotland. Experts represented a range of professional bodies including AGILE-the Chartered Society of Physiotherapy Older People’s Network, the British Geriatrics Society, College of Occupational Therapists Older Peoples Specialist Section, and Admiral Nurses specialising in dementia care. 16 experts completed the 4 rounds of the modified Delphi process.

### Summary of rounds

The results for each round are presented in Table 1. This table presents an overview of the scoring for each round and outlines the following:

• Total number of statements that participants were asked to score and comment on

• Statements that reached 70% agreement and were accepted in each round

• Statements that did reach the 70% consensus however comments indicated the wording changes would improve the clarity and were therefore revised and included in the following round to be rescored

• Statements that were removed as they did not reach consensus and the free text comments supported removal of the statement

• Statements that did not reach consensus however comments suggested wording changes to improve clarity and included in the following round to be rescored

• New statements that were generated from the free text comments and suggestions from participants

**Table 1:** **Summary of responses Table**2**accepted statements**

	**Total number of statements for scoring**	**Primary aim of round**	**Statements that reached consensus (< 70%) and were accepted**	**Statements that reached consensus but were revised based on comment and rescored in next round**	**Statements that were removed**	**Statements that did not reach consensus and were modified**	**New statements generated from comments**
Round 1	42	Exploratory	22 (52%)	6	3	11	5
Round 2	22	Exploratory and clarifying	16 (73%)	0	0	6	4
Round 3	10	Exploratory and clarifying	4 (40%)	0	2	4	0
Round 4	4	Confirmatory	4 (100%)	0	0	0	0

In round one, 22 statements were accepted, 3 were removed, 5 new were inserted, and 17 were modified. In round two, 16 statements were accepted, none were removed, 4 new were inserted and 6 were modified. In round three, 4 statements were accepted, 2 were removed, no new were inserted and 4 were modified. In round four, 4 statements were accepted and none were removed or modified and no new ones were inserted.

### Summary of results

Consensus was reached on forty-six statements relating to seven domains of chair based exercise: definition, intended users, potential benefits, structure, format, risk management and evaluation. These domains were identified by the initial telephone consultations and the team workshop of the study management group in the development of the framework for the process.

All statements that reached consensus are presented in Table 2 within the seven domains.

**Table 2:** Accepted statements

	**Statement**	**% of agreement**	**When was consensus reached**
Definition	CBE should be considered as part of a continuum of exercise for frail older people where progression is encouraged	100	Round 2
	CBE should be used flexibly to respond to the changing needs of frail older people	100	Round 2
	The purpose of using a chair is to promote stability in both sitting and standing	87.5	Round 2
	Where possible CBE should be used as starting point to progress to standing programmes	76.5	Round 1
	CBE is primarily a seated exercise programme	75	Round 2
Intended users	CBE can be considered as part of a progressive falls exercise pathway with the aim of progressing to evidence based standing programmes	93.75	Round 2
	For use with older people who are unable to carry out standing exercises as a consequence of an acute medical problem from which they might improve and progress to weight bearing exercises	88.2	Round 1
	For use with older people with an activity limitation who cannot participate in other forms of exercise	76.5	Round 1
Potential benefits	If tailored appropriately CBE can be beneficial in improving the following:		
	- mood and well-being	100	Round 1
	- certain activities of daily living	93.75	Round 2
	- mobility around joints	93	Round 3
	- social interaction	88.2	Round 1
	- muscle strength	88.2	Round 2
	- certain personal activities of daily living	87.5	Round 2
	- co-ordination	78.25	Round 3
	- confidence with activities of daily living	70.6	Round 1
Structure	The delivery of sessions and exercises can be tailored to individual preference within a structured programme	93.75	Round 2
	All CBE programmes should include progressive resistance training that is tailored to the individual	93.7	Round 2
	Each session should begin with an appropriate warm up	88.2	Round 1
	Music can be beneficial as part of programmes if used appropriately and it is welcomed by participants	87.5	Round 2
	Strength training can include the use of resistance bands, weights and body weight resistance exercises	87.5	Round 2
	Cardiovascular interval training should be performed to prevent fatigue, if appropriate and tailored	87.5	Round 4
	Participants should be encouraged to work at an intensity which is appropriately challenging for them	86	Round 3
	Each session should include developmental stretches	82.3	Round 1
	Each sessions should end with an appropriate cool down	82.3	Round 1
	Each session should include a component of strength resistance training, endurance training and cardiovascular fitness	76.3	Round 1
	Strength training should be targeted to meet nominated programme aims	76.3	Round 1
	Cardiovascular training should be performed at a moderate intensity for all participants	76.3	Round 1
Format	Each session should be carried out at least once a week	94.1	Round 1
	Rolling programmes are appropriate with new participants joining at any point	94.1	Round 1
	Gradually building up the duration of sessions can be beneficial for fail older adults with reduced exercise tolerance	93.75	Round 2
	Each session should last no longer than an hour	88.2	Round 1
	Programmes should be tailored to meet individual needs	88.2	Round 1
	The goal of CBE should be clearly defined for each individual participant	88.2	Round 1
	The number of CBE sessions should be tailored to the individual needs of the participants	81.25	Round 2
	Each CBE session should be a minimum of 10 minutes long with a view to increasing further	75	Round 4
Risk management	All programmes should be run by a suitably skilled and trained leader	100	Round 1
	Instructors should have knowledge and skills of working with frail older people	100	Round 1
	Programmes do not have to be delivered by healthcare professionals	94	Round 1
	An individual health assessment should be carried out prior to commencing a CBE programme	93.75	Round 4
	Instructors should be aware of medical conditions which could disqualify participation or which require careful monitoring throughout sessions on the grounds of safety	87.5	Round 2
	CBE training programmes need to be regulated to ensure that they meet the agreed training curriculum	86	Round 3
	All instructors should have completed a regulated CBE training programme	81.25	Round 4
Evaluation	Participants of CBE should be encouraged to actively feedback on each session	100	Round 3
	Participant reported outcome measures are useful for evaluating the effectiveness of programmes	94.1	Round 1
	If CBE’s are undertaken for health gains, a standardised outcome measure should be used routinely throughout programmes to evaluate effectiveness	70.6	Round 1

5 statements were removed throughout the process due to not reaching the threshold for consensus and the thematic analysis of the free text responses. One statement was removed from the definition domain in Round 3, one statement was removed in the intended users’ domain in round 1, two statements were removed in the potential benefits domain in rounds 1 and 3 and 1 statement was removed in the structure domain in round 1. All statements that were removed are presented in Table 3.

**Table 3:** Removed statements

	**Statement**	**% of agreement**	**Outcome**	**Selected comments**
Definition	Chair based exercise can include static standing exercises (e.g. sit-stand). Once dynamic standing exercises are included this is no longer considered chair based exercise	68.75	Removed following Round 3	‘I wouldn’t describe sit-stand as static’ ‘unsure whether static is the correct word to use’
				‘Do you mean by static that both feet remain in a fixed position’ ‘a chair was designed to sit in, and stand up from- beyond that we are stretching the purpose’
Intended users	Encouraged for older people who are concerned about stability in movement	64.71	Removed following Round 1	‘…most older people have some concern about stability yet for most by doing standing work this will improve’
Potential benefits	Chair based exercise is beneficial for reducing pain	52.94	Removed following Round 1	‘Depends on the source of the pain’
				‘Is there any evidence relative to pain management?’
	Chair based exercise is beneficial for improving ambulation	68.75	Removed following round 3	‘Think we have to be very careful, if CBE is CBE (i.e., seated) therefore is not going to improve standing activity’
Structure	Chair based exercise programmes should ideally be carried out in a group environment	52.94	Removed following Round 1	‘neutral because some people will not want to be in a group and others will not be able to get to a group’
				‘Group environments are best as the social interaction can be a vital component of adherence and motivation - however home exercises can be just as effective if carried out correctly and maybe with supervision’

From the accepted statements at the end of the modified Delphi process the study management group constructed a definition of CBE which was emailed to the Delphi panel. This definition was modified following minor comments from the Delphi panel and the final definition was approved by 14 of the 16 Delphi panel experts (87.5%).

Chair based exercise has been defined by this process as:

“a primarily seated, structured and progressive exercise programme that is part of a continuum of exercise for older people, which uses a chair to provide stability, and is delivered by instructors that are suitably skilled and trained to work with frail older people”.

## Discussion

This study aimed to utilise expert opinion to define chair based exercise for older people and develop a core set of principles for chair based exercise programmes. Consensus was reached on forty-six statements relating to seven domains of chair based exercise: defining, intended users, potential benefits, structure, format, risk management and evaluation. This work has clinical value in offering the first efforts at defining CBE and for providing greater clarity and understanding on the role and scope of CBE for older people.

The complexities of CBE as an intervention have been highlighted with a clear agreed definition of CBE proving challenging. Several rounds of consultation were required to reach any level of agreement regarding the scope and purpose of CBE for older people. It is clear that many experts do not want CBE to be regarded as the default exercise programme for all older people without appropriate justification and progression. These concerns are reflected by the expert discussions with a reluctance to commit to predefined and prescriptive amount of seated activity within a programme. Instead comments centred on the need to consider appropriate progression to more challenging and dynamic standing programmes to maximise health benefits. The definition established in this process does however offer a framework for CBE outlining that it is primarily a seated programme that uses a chair to provide stability. This framework allows CBE to be adapted to meet the changing needs of older people in the appropriate setting and perhaps a more prescriptive model would have limited applicability in health and social care settings.

CBE has been used as a control in research studies testing exercise interventions often described as low level exercise [16] suggesting CBE may be viewed as a default option for frail older people with limited effectiveness. This consensus process however suggests that CBE is appropriate for older people who are unable to take part in other forms of exercise due to activity limitation which may be acute or longer term. Experts predominantly working in the healthcare sector stressed the importance of progression for all users of CBE – whether it is to standing exercise programmes or a progressively challenging seated programme. Progressively challenging programmes are supported by evidence of effective exercise for frail older people [17] and identify that if delivered appropriately CBE can be underpinned by the principles of exercise for frail older people [18].

The reasons for promoting CBE to older people varied amongst the expert panel and included both health and social gains; however it was acknowledged that there should be a clearly defined goal for each participant. The predominant reason for participants to attend needed to be acknowledged and would influence the programme content and outcome. Formally measuring improvements and facilitating progression may not be justified for those users who choose to attend CBE group for primarily social reasons.

It could be argued that the distinction needs to be made between when CBE is being undertaken for specific health gains and when it is the exercise of choice for an older person. It is however essential that older people are fully informed about CBE to reduce any misconceptions over the potential benefits and reasons for taking part.

Exercise has been shown to have wide ranging health benefits for older people and the potential benefits offered by CBE are in line with this evidence [19]. It is however important to recognise for some intended users of CBE for example those with a chronic long term condition the likely outcomes may be different. From a physiological perspective CBE may offer a way of protecting against the progression of musculoskeletal frailty rather than having a restorative role. Comments relating to maintaining functions were identified by experts however the agreed principles do not reflect this role of CBE.

The outputs from the Delphi suggest that the duration of exercise sessions should be of 10 minutes or longer, using progressive resistance (strength) training. This is broadly in line with the UK national exercise guidance for older people [20]. The consensus achieved here differs from the national guidance by stating that a minimum programme should be one session per week, lasting up to an hour, whilst national recommendations require programmes to achieve 150 minutes per week over two or more sessions [20]. However, these national guidelines are generic, covering all types of exercise in a variety of patients, whilst CBE, as defined through this study, is useful for those who cannot participate in other forms of exercise and therefore a less intensive approach in this context may be reasonable. Other advice, such as from the BHF National Centre for Physical Activity and Health [21], supports lower intensity programmes in patients unable to tolerate higher intensity exercise with a view to progressing intensity and duration as able.

People participating in chair based exercise programmes may well be sedentary for much of the time when they are not doing exercise, and so the potential benefits of chair exercise may be offset by the hazards of inadequate customary physical activity at other times. Thus, although the consensus statements have defined a programme that might be expected to achieve worthwhile health benefits, doubt remains as to whether these will be demonstrable in practice, or without additional measures to increase physical activity between exercise sessions.

Managing the potential risks of CBE programmes was considered important by the expert panel and ensuring appropriate training and regulation was commented on throughout, often being raised in domains outside risk management.

Consensus was however not reached on a minimum qualification required for instructors as this impacted on the volunteers and support workers that are currently trained to deliver programmes. Instead agreement was reached on having regulated CBE training programmes that follow an agreed curriculum to improve standardisation and quality. It is acknowledged that health professionals and instructors influence the attitudes and beliefs of older people to exercise [22] and there is evidence to suggest that instructors have different attitudes depending on the setting they work and whether they predominantly deliver seated or standing programmes [23]. A greater focus on the training for instructors which encourages positive attitudes towards seated programmes is essential to ensure that older people are able to take part in programmes that are delivered by well-trained and motivated instructors and who have the appropriate skills to deliver programmes in line with the established principles.

The findings of this study are of value as a guide to practitioners involved in the delivery of exercise for older people and for those who purchase these services. They provide a benchmark against which current practice can be evaluated. The outputs of this study also provide evidence of clear hypothesis driving chair-based exercise, in terms of its objectives and likely benefits. Evaluative research is now possible to evaluate the effectiveness and cost effectiveness of CBE against these stated goals. The broad consensus achieved here provides much needed clarity for both practitioners and researchers.

### Strengths and limitations

The consensus threshold of 70% achieved the desired effect of allowing us to complete our process, without participant drop-out, within four iterations. Agreement for most statements exceeded 70% by some degree. However, at this threshold, our findings must be taken as the best achievable consensus given the current lack of robust evidence in the field, rather than as evidence of absolute unanimity.

The focus and findings of this study were influenced by the perspectives of both the research team and consulted experts, who were clinicians and experts in the delivery of chair based exercise. The framework for round one was developed by the research team in consultation with experts which will have influenced the scope of the process. This modified Delphi technique may have introduced bias from experts when rating the structured statements in round one. Experts may have responded more favourably to the predefined statement rather than to iterate a statement of their own. Nevertheless only 52% of statements were accepted in round one and opportunity was given for experts to freely comment on all statements and suggest new themes and statements.

In addition experts in exercise for older people may not be entirely impartial in their appraisal of chair based exercise as they have already invested and engaged with the concept which may have led to more favourable responses.

However, despite these limitations we consider that our use of the Delphi process provided a fair representation of the expert practitioner view of chair based exercise, as we consulted a mixed group of experts spanning health and social care, voluntary and private sector groups as well as academia. The established principles offer a framework for CBE programmes that requires further evaluation in terms of its acceptability, feasibility and effectiveness.

## Conclusions

A definition for CBE for older people has been suggested based on clinical expert opinion which has previously been lacking in the literature. Agreement has been reached on a set of principles of CBE for older people through a Delphi process with a mixed group of experts.

## Competing interests

The authors declare that they have no competing interests.

## Authors’ contributions

KR co-ordinated the running of the study, recruited participants, contributed to the formation of the Delphi statements and between round analysis and preparation of the manuscript. PLeighton provided input into the qualitative analysis and contributed to the preparation of the manuscript. PLogan, KA, AG and JG were part of the study management group contributing to the development of the Delphi statements, between round analysis and preparation of the manuscript. TM acted as supervisor to KR and part of the study management group contributing to the development of the Delphi statements, between round analysis and preparation of the manuscript. All authors read and approved the final manuscript.

## Pre-publication history

The pre-publication history for this paper can be accessed here:

http://www.biomedcentral.com/1471-2318/14/65/prepub

## Expert panel members: expertise and affiliations

Lorraine Butterworth: Admiral Clinical Lead Nurse, Cornwall Care.

Irene Grainger: Senior Physiotherapist (Falls and Dementia), Hope Specialist Service, Grimsby, North East Lincolnshire.

Dr Victoria Goodwin: Senior Research Fellow and Physiotherapist, University of Exeter Medical School.

Simon Hanna: Clinical Exercise Specialist, Cambridgeshire Community Services.

Dr Helen Hawley- Hague: Research Associate, University of Manchester.

Victoria Johnston: Senior Physiotherapist, Chair of AGILE (Chartered Physiotherapists working with Older People).

Bob Laventure: Director Later Life Training and Older People Lead, BHF National Centre for Physical Activity and Health, Loughborough University

Tracy Levy: Exercise Instructor, EXTEND Exercise Training.

Edith Macintosh: Rehabilitation Consultant, Care Inspectorate, Scotland.

Dr Gladys Onambélé-Pearson Department of Exercise & Sport Science, Manchester Metropolitan University, UK.

Bhanu Ramaswamy: MCSP Independent Physiotherapy Consultant and Visiting Fellow Sheffield Hallam University.

Pasna Sallis: Specialist Occupational Therapist, NHS Greater Glasgow and Clyde and College of Occupational Therapy Specialist Section- Older People

Professor Dawn Skelton: Exercise Physiologist, Institute of Applied Health Research, Glasgow Caledonian University.

Anne Thomson: Visiting Lecturer in Physiotherapy, Chair of Trustees EXTEND Exercise Training.

Janet Thomas: Senior Physiotherapist, AGILE (Chartered Physiotherapists working with older people) and Later Life Training Tutor.

Bex Townley: Later Life Training & L4 Exercise Specialist Hywel Dda Health board.

Lawrence Wintergold: Professional Lead Occupational Therapist, Older Peoples Services, Sussex Partnership NHS Foundation Trust.

## References

[B1] Spirduso W Francis K MacRae P Physical dimensions of aging (Ed. II) 2005 Illinois: Human Kinetics

[B2] Sherrington C Whitney JC Lord SR Herbert RD Cumming RG Close JCT Effective exercise for the prevention of falls: a systematic review and meta-analysis J Am Geriatr Soc 2008 14 12 2234 2243 10.1111/j.1532-5415.2008.02014.x 19093923

[B3] Campbell AJ Robertson MC Gardner MM Norton RN Tilyard MW Buchner DM Randomised controlled trial of a general practice programme of home based exercise to prevent falls in elderly women BMJ 1997 14 315 10.1136/bmj.315.7115.1065 PMC2127698 9366737

[B4] Baum E Jarjoura D Polen AE Faur D Rutecki G Effectiveness of a group exercise programme in a long term care facility: a randomized pilot trial J Am Dir Assoc 2003 14 2 74 80 10.1016/S1525-8610(04)70279-0 12807578

[B5] Anthony K Robinson K Logan P Gordon A Harwood R Masud T Chair based exercises for frail older people: a systematic review Biomed Res Int 14 Available online at http://www.hindawi.com/journals/bmri/2013/309506/10.1155/2013/309506 PMC3782120 24089670

[B6] Hasson F Keeney S McKenna H Research guidelines for the Delphi survey technique J Adv Nurs 2000 14 4 1008 1015 11095242

[B7] Williams PL Webb C The Delphi technique: a methodological discussion J Adv Nurs 1994 14 180–6BMJ 1995: 311-376 8138622 10.1111/j.1365-2648.1994.tb01066.x

[B8] Keeny S Hasson F McKenna HP A critical review of the Delphi technique as a research methodology for nursing Int J Nurs 2001 14 2 195 200 10.1016/S0020-7489(00)00044-4 11223060

[B9] Mullen PM Delphi myths and reality J Health Organ Manag 2003 14 1 37 52 10.1108/14777260310469319 12800279

[B10] Rowe G Wright G Expert opinions in forecasting; The Role of the Delphi technique Int Ser Oper Res Manage Sci 2001 14 125 10.1007/978-0-306-47630-3_7

[B11] Sumison T The Delphi technique: an Adaptive Research Tool Br J Occup Ther 1998 14 4 153 156

[B12] Vernon W The Delphi technique: a review Int J Ther Rehabil 2009 14 2 69 76

[B13] Diamond IR Grant RC Feldman BM Pencharz PB Ling SC Moore AM Wales PW Defining consensus: a systematic review recommends methodological criteria for reporting of Delphi studies J Clin Epidemiol 2014 14 4 401 409 10.1016/j.jclinepi.2013.12.002 24581294

[B14] van Steenkiste BC Jacobs JE Verheijen NM Levelink JH Bottema BJ A Delphi technique as a method for selecting the content of an electronic patient record for asthma Int J Med Inform 2002 14 1 7 16 10.1016/S1386-5056(01)00223-4 11904244

[B15] Wells C Kolt GS Marshall P Bialocerkowski A The definition and application of Pilates Exercise to Treat People with Chronic Low back pain: a Delphi Survey of Australian Physical Therapists Phys Ther 2013 doi:10.2522 10.2522/ptj.20130030 24179139

[B16] Au-Yeung S Ho H Lai J Lau R Wong A Lau SK Did Mobility and Balance of Residents Living in Private Old Age Homes Improve After A Mobility Exercise Programme? A Pilot Study Hong Kong Physiotherapy J 2002 14 16 10.1016/S1013-7025(09)70027-2

[B17] Hruda K Hicks A Mccartney N Training for Muscle Power in Older Adults: Effects on Functional Abilities Can J Appl Physiol 2003 14 2 178 189 10.1139/h03-014 12825328

[B18] Chin A Paw MJ van Uffelen JG Riphagen I van Mechelen W The functional effects of physical exercise training in frail older people Sports Med 2008 14 9 781 10.2165/00007256-200838090-00006 18712944

[B19] Taylor D Physical Activity is Medicine for Older Adults Postgrad Med J 2014 14 26 32 10.1136/postgradmedj-2012-131366 24255119 PMC3888599

[B20] Department of Health Start Active, Stay Active- A report on physical activity from the four home countries Medical Officers 2011 Department of Health London Available online at https://www.gov.uk/government/publications/start-active-stay-active-a-report-on-physical-activity-from-the-four-home-countries-chief-medical-officers

[B21] British Heart Foundation National Centre for Physical Activity and Health Interpreting the UK Physical Activity Guidelines for Older People: Guidance for Those who Work With Frailer, Older People 2012 British Heart Foundation National Centre for Physical Activity and Health, Loughborough University Available at: http://www.bhfactive.org.uk/older-adults-resources-and-publications-item/39/430/index.html

[B22] Hawley H Older adults’ perspectives on home exercise after falls rehabilitation—an exploratory study Health Educ J 2009 14 3 207 218 10.1177/0017896909339533

[B23] Hawley H Skelton D Campbell M Todd C Are the attitudes of exercise instructors who work with older adults influenced by training and personal characteristics? J Aging Phys Act 2012 14 1 47 63 22190119 10.1123/japa.20.1.47

